# Can kids identify unprocessed fruit as healthier than an ultra-processed sugar-sweetened beverage? Functional versus self-reported nutrition knowledge and dietary intake among youth from six countries: findings from the International Food Policy Study

**DOI:** 10.1186/s40795-025-01109-y

**Published:** 2025-07-01

**Authors:** Liza Boyar, Christine M. White, Lana Vanderlee, Jasmin Bhawra, David Hammond

**Affiliations:** 1 School of Public Health Sciences, University of Waterloo, 200 University Avenue West, Waterloo, ON Canada; 2 École de nutrition, Centre NUTRISS, Université Laval, 2440 boulevard Hochelaga, Québec, QC Canada; 3 School of Occupational and Public Health, Toronto Metropolitan University, 350 Victoria St, Toronto, ON Canada

**Keywords:** Food, Youth, Nutrition knowledge, Dietary intake

## Abstract

**Background:**

Consumption of ultra-processed foods is associated with a range of poor dietary and health outcomes. Although lower nutrition knowledge is associated with higher consumption of ultra-processed foods, few studies have directly compared nutrition knowledge among youth from countries with different food environments and nutrition policies. This study examined whether youth could identify differences in nutritional quality between a commonly consumed ultra-processed and unprocessed food.

**Methods:**

Cross-sectional surveys were conducted with youth aged 10–17 (*n* = 12,489) from Australia, Canada, Chile, Mexico, United Kingdom (UK), and United States (US) as part of the 2020 International Food Policy Study. Participants were shown images of two products in random order, corresponding to “unprocessed or minimally processed” (apple) and “ultra-processed” (apple fruit drink) foods under NOVA classification system, and asked to rate the healthiness of each. Respondents who rated the apple higher than the apple fruit drink were assigned a “correct” score. Regression models examined differences in “correct” responses by country, perceived nutrition knowledge, perceived diet healthiness, intake of fruits/vegetables, and intake of less healthy foods.

**Results:**

Mexican (96.5%) and Chilean (94.3%) youth were most likely to correctly identify the unprocessed apple as “healthier” than the ultra-processed apple fruit drink, whereas US youth were the least likely (79.6%, *p* < 0.001 for all). Perceived nutrition knowledge was inversely associated with correct scores (*p* < 0.001). Youth who reported the highest (AOR: 0.43, *p* < 0.001) and lowest (AOR: 0.57, *p* < 0.05) categories of perceived diet healthiness had the lowest odds of correct responses. Higher intake of both less healthy foods (AOR: 0.70, *p* < 0.001) and fruits/vegetables (AOR: 0.87, *p* < 0.001) were associated with lower odds of correct responses.

**Conclusions:**

Across countries, 5–20% of youth were unable to correctly identify an unprocessed fruit as ‘healthier’ than an ultra-processed fruit drink, with notable country differences. Further research is needed to examine differences for a broader range of foods and levels of processing. Education campaigns should ensure that young people have basic knowledge about the relative dietary quality of commonly consumed foods, particularly in the US. Discrepancies between perceived and objective nutrition knowledge additionally highlight the need for objective measures of knowledge to be included in assessments.

**Supplementary Information:**

The online version contains supplementary material available at 10.1186/s40795-025-01109-y.

## Background

Childhood is an important life stage for the development of healthy nutrition behaviours [1]. Early nutrition behaviours have been shown to persist into adulthood, with unhealthy dietary habits continuing into later life [2–4]. This is particularly important in light of the rise in non-communicable diseases among young people, including type 2 diabetes and obesity, which point to youth’s nutrition behaviour as a pivotal predictor of morbidity and mortality in adulthood [4–7].

Diet quality among youth is determined by a wide range of factors, including the food environment, social, and familial factors [8–11]. Within this broader context, dietary intake and nutrition behaviour are closely associated with nutrition knowledge in youth [12, 13]. In particular, higher nutrition knowledge has been associated with increased fruit, vegetable, and fish consumption, while lower nutrition knowledge has been associated with a higher consumption of desserts, snacks, and fried foods [1, 13, 14]. Accordingly, nutrition policies in many countries include education and school-based interventions to increase nutrition knowledge and promote healthy diets among young people [15].

Nutrition knowledge has been conceptualized in many different ways, ranging from a focus on specific nutrients and their relevance to health, to broader concepts such as food preparation skills and awareness of national dietary recommendations [9, 11, 16, 17]. In some cases, nutrition knowledge is used interchangeably with nutrition literacy and food literacy, which refer to the ability to broadly understand, identify and apply factual nutrition information [16, 17]. A recent systematic review demonstrated that most studies on nutrition knowledge use questionnaires which ask youth about a variety of topics including healthy eating, food groups, roles of nutrients in the body, serving sizes, food skills, and relationship of nutrients to diseases [13]. This acts as a measure of perceived nutrition knowledge; in contrast to functional knowledge, which gauges an individual’s ability to not just understand, but also to apply knowledge in identifying healthier foods, resulting in a stronger correlation with dietary behaviour [18]. It is also common for studies to focus on the relationship between youth’s dietary behaviours and parental nutrition knowledge, rather than the knowledge of the youth themselves [3, 14]. Differences in the way in which nutrition knowledge has been conceptualized and measured make it difficult to compare nutrition outcomes at the population-level over time, between countries, or across studies [8, 17], particularly for nutrition knowledge among young people [11, 13, 14].

One component of nutrition knowledge that has gained attention is consumers’ understanding of levels of food processing. Food processing level is commonly classified using the NOVA food classification system, which divides foods into four groups according to the extent and purpose of the industrial processing: unprocessed or minimally processed foods, processed culinary ingredients, processed foods, and ultra-processed foods [19]. Ultra-processed foods tend to be high in saturated fat, salt, and added sugar, as well as low in fibre and vitamins [20, 21]. Greater intake of ultra-processed food has previously been associated with lower diet quality and greater risk of nutrition-related diseases among adults [20, 22–25]. Youth are the leading consumers of ultra-processed foods globally, making up 30–60% of their total daily energy intake [20, 26–29]. Research on the associations of ultra-processed food consumption and excess adiposity in the pediatric population is mixed [30–32]; however, there is evidence to suggest that lower consumption of ultra-processed foods high in saturated fat, salt, and sugar is associated with better diet quality in youth [33–36]. Recommendations to limit ultra-processed foods have thus been incorporated into national dietary guidelines in several countries, including Canada, Brazil, Chile, and Mexico [20, 37–39]. Accordingly, the ability to identify unprocessed or minimally processed foods as ‘healthier’ than ultra-processed foods is an important aspect of nutrition knowledge.

Previous studies have assessed whether consumers perceived differences in nutrition quality of foods based on level of food processing [40]. Additionally, although nutrition knowledge has been measured in various countries [1, 3, 5, 12, 14], to our knowledge, only one published study has compared youth’s nutrition knowledge across countries using consistent measures [41]. Specifically, the HELENA (Healthy Lifestyle in Europe by Nutrition in Adolescence) study was conducted once in ten urban European cities in 2006–2007 [42] and found significant differences in functional nutrition knowledge between countries, but did not describe the nature of the difference as country comparison was not an objective of that study [41]. Overall, few studies have directly compared nutrition knowledge among youth from countries with different food environments and nutrition policies. Thus, the current study sought to examine whether youth could identify differences in the nutritional quality or “healthiness” of a commonly consumed ultra-processed sugar-sweetened beverage (SSB, an apple-flavored fruit drink) and an unprocessed food (an apple) – a functional measure of nutrition knowledge. The study had four primary objectives, including examining: (1) country-level differences in functional nutrition knowledge among youth in Australia, Canada, Chile, Mexico, United Kingdom (UK), and United States (US); (2) differences in functional nutrition knowledge by demographic characteristics (age, sex-at-birth, ethnicity, perceived income adequacy, and Body Mass Index (BMI)); (3) associations between functional nutrition knowledge and perceived nutrition knowledge; and (4) associations between functional nutrition knowledge and perceived diet healthiness and dietary intake of healthy and less healthy foods.

## Methods

Data were collected as part of the 2020 International Food Policy Study (IFPS) Youth Survey, a cross-sectional survey conducted in Australia, Canada, Chile, Mexico, UK, and US. Data were gathered via self-completed web-based surveys conducted in November-December 2020 with youth aged 10 to 17 years. Respondents were recruited through parents/guardians enrolled in the Nielsen Consumer Insights Global Panel and their partners’ panels. Email invitations with unique survey links were sent to a random sample of adult panelists within each country. Those who confirmed they had a child aged 10 to 17 living in their household, for whom they were the parent/legal guardian, were asked to provide informed consent for their child to complete the survey (only one child per household was invited). Youth aged 10 to 17 years were eligible to participate, with quotas for age and sex-at-birth groups in the UK and US. A larger sample was recruited in Canada than the other countries to provide greater power for sub-national tests between provinces unrelated to the current analysis. The American Association for Public Opinion Research cooperation rate #2 was 79.6% in 2020, calculated as the percentage of participants who completed the survey out of eligible participants who accessed the survey link [43].

After eligibility screening, all potential youth respondents were provided with information about the study and asked to provide assent before beginning the survey. Surveys were conducted in English in Australia and the UK; English or French in Canada; Spanish in Chile and Mexico; and English or Spanish in the US, with adaptations for country-specific terminology. Members of the research team who were native in each language reviewed the French and Spanish translations independently. The child’s parent/guardian received remuneration in accordance with their panel’s usual incentive structure (e.g., points-based or monetary rewards, chances to win prizes). This study was conducted according to the guidelines laid down in the Declaration of Helsinki and all procedures involving human subjects/patients were reviewed by and received ethics approval through a University of Waterloo Research Ethics Committee (ORE# 41477). Written assent was obtained from all participants. A full description of the study methodology, including survey details can be found in the 2020 International Food Policy Study Technical Report [44]. The conceptual framework of the study has likewise been published [45].

### Measures

#### Functional nutrition knowledge

**Fig. 1:**
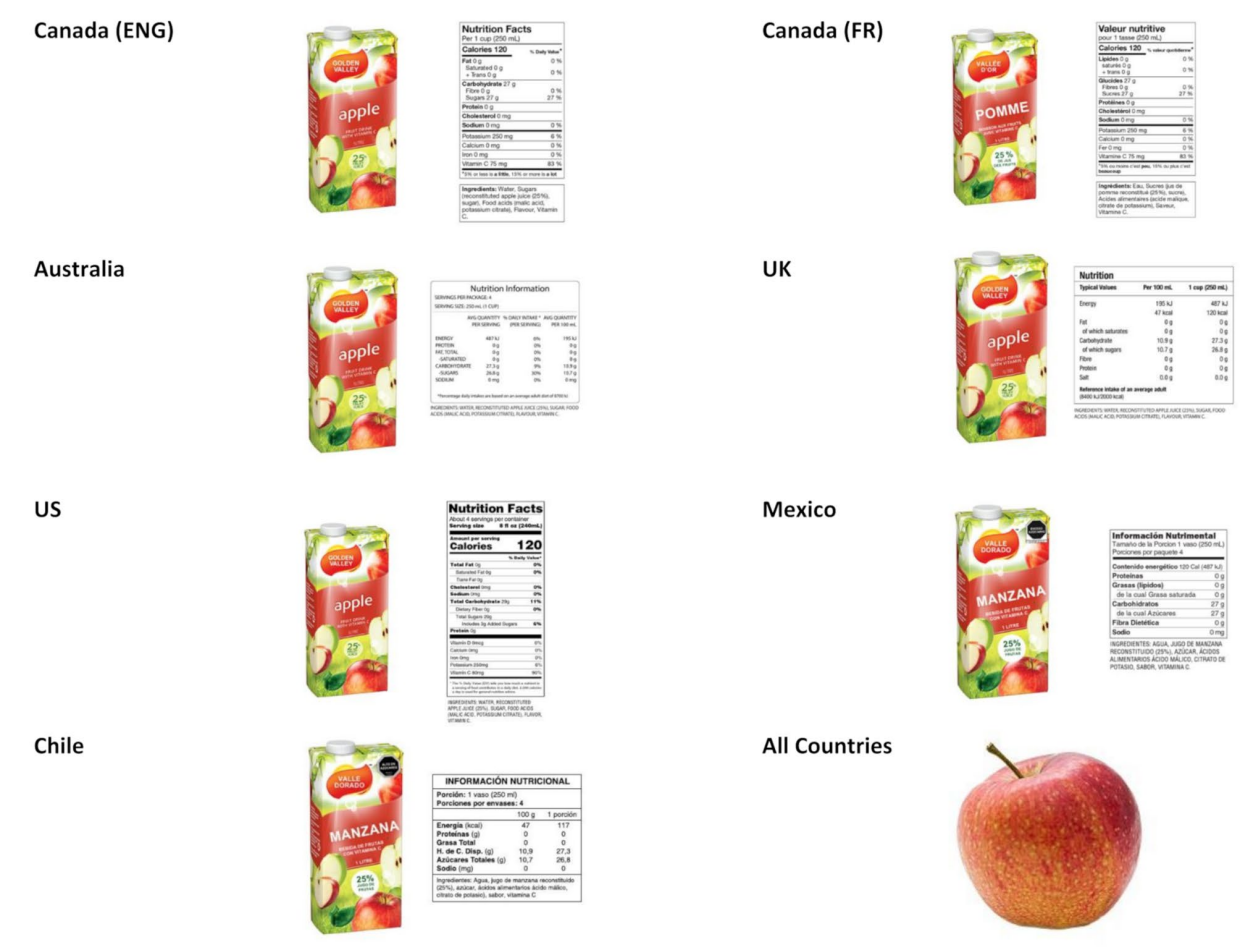
Images of apple and apple fruit drink, as displayed in each country survey

Functional nutrition knowledge was assessed by showing participants an image of an apple (a Group 1 or ‘unprocessed’ food under the NOVA classification system), and an image of a fictitious brand of apple fruit drink (a Group 4 or ‘ultra-processed’ food) [19, 46]. The images of the two products were shown one at a time, in random order. The image of the apple fruit drink was accompanied by country-specific nutrition facts tables in all countries, and front-of-pack ‘high-in sugar’ warning labels in Chile and Mexico, as the drink exceeded the warning label threshold for sugar (see Fig. 1). No nutrition facts table was included for the apple as fresh fruit is exempt from displaying a nutrition facts table. Participants were asked to rate each product on a scale of 0 to 10, where 0 = “not at all healthy” and 10 = “extremely healthy”. Respondents who ranked the apple higher than the fruit drink on the healthiness scale were assigned a ‘correct’ score; respondents who reported higher or equivalent scores for the fruit drink were assigned an ‘incorrect’ score. Participants with missing data due to “refuse to answer”, and “don’t know” responses on either rating were excluded from the analysis. Respondents could complete the survey on smartphones, tablets, or computers, with the scale formatted vertically on smartphones for improved usability/easier scrolling.

#### Perceived nutrition knowledge

Perceived nutrition knowledge was assessed by asking, “How much do you know about healthy eating and nutrition?” using a 0 to 10 scale, where 0 = “nothing” and 10 = “a lot”. Responses were recorded and analyzed as quartiles wherein 0–5 = “very low”, 6–7 = “low”, 8 = “moderate”, and 9–10 = “high”.

#### Perceived diet healthiness

Self-reported diet quality was assessed by asking, “Overall, how unhealthy or healthy is the food you usually eat?”. Response options ranged from “very unhealthy” to “very healthy” on a 5-point Likert scale.

#### Dietary intake

Frequency of fruit/vegetable intake was measured by asking “How many times did you eat fruit yesterday?” and “How many times did you eat vegetables yesterday?”, with response options for each ranging from “0” to “10 or more”. Participants were also asked whether or not they had sugary drinks, fast food from a restaurant, sugary cereals, snacks (e.g., crackers, chips or granola bars), or desserts yesterday. Responses were recoded to binary (1 = ‘consumed yesterday’, 0 = ‘not consumed yesterday’) and summed together to create an index of less healthy food intake (range 0–5). These dietary intake measures were adapted from existing measures [47], and have undergone cognitive testing to ensure comprehension and ease of use.

#### Demographic characteristics

Demographic measures included sex-at-birth (Male/Female), age (10–13/14–17), ethnicity, perceived income adequacy, and BMI. Ethnicity was assessed using country-specific race or ethnicity categories and analysed as a derived variable (Majority/Minority/Unstated) to accommodate different measures across countries. Majority constituted the following ethnicities: in Canada if “White (European descent)” was the only category selected; in Australia if only speak English at home; in UK and US if only the “White” category was selected; in Mexico and Chile if did not consider self Indigenous. Perceived income adequacy was assessed with the question, “Does your family have enough money to pay for things your family needs?” (Not enough money/Barely enough money/Enough money/More than enough money/Unstated) [48]. BMI categories were derived using self-reported height and weight, and classified using the WHO 2007 growth reference for children and adolescents BMI-for-age z-scores by sex where severe thinness: z-score<-3, thinness: -3 < = z-score<-2, normal weight:-2 < = z-score < = 1, overweight: 1 < z-score < = 2, obesity: z-score > 2 [49, 50]. Due to low cell sizes, the categories for “severe thinness” and “thinness” were combined. Participants with missing responses, including those missing because the respondent selected “Don’t know” or “Refuse to answer” and extreme values (BMI-for-age z-score<-5 or z-score > 5), were retained and grouped together as a separate category in analyses. This was done as individuals who choose not to self-report weight and/or height may differ from those who respond; those with larger body size are more likely to underreport or refuse to report either their height or weight particularly given the heightened potential for social desirability bias when responding to a survey on diet and nutrition [51].

### Data analysis

A total of 12,489 youth completed the survey in 2020. Respondents were excluded for the following reasons: region was missing, ineligible or had an inadequate sample size (i.e., Canadian territories); invalid response to a data quality question; survey completion time under 10 min; and/or multiple invalid responses to open-ended measures (*n* = 458). A sub-sample of 11,232 (Australia: *n* = 1,474; Canada: *n* = 3,601; Chile: *n* = 1,498; Mexico: *n* = 1,724; UK: *n* = 1,426; US: *n* = 1,509) were included in the current analysis after excluding 799 further respondents with missing data in at least one of the following six measures of interest: rating of apple/apple fruit drink healthiness, perceived nutrition knowledge, perceived healthiness of diet, diet intake (fruit/vegetables frequency, consumption of sugary drink/fast food/cereal/snack/dessert), ethnicity, and perceived income adequacy. Specifically, 27 participants were missing data on the apple rating, 208 participants were missing data on the apple fruit drink rating, and 13 participants were missing data on both item ratings. Though frequency of missing data was greater for the apple fruit drink than the apple, it represents a relatively small proportion of the total sample. Overall, 2.1% of the sample (*n* = 248) was excluded due to missing/invalid data on the rating of apple/apple fruit drink healthiness, the primary outcome of interest; this included 2.1% in Australia, 2.5% of respondents in Canada, 3.0% in Chile, 1.1% in Mexico, 1.8% in UK, and 1.3% in the US.

Data were weighted with post-stratification sample weights constructed using a raking algorithm with population estimates from the census in each country based on age group, sex-at-birth, region, and ethnicity (except in Canada). Four models were constructed. First, a binary logistic regression model examined correlates of correct responses (where 0 = ‘incorrect’ and 1 = ‘correct’). The model was run in two steps: (1) a main effects model; and (2) two-way interaction terms between country and each demographic variable to examine potential demographic differences across countries. Next, three separate binary logistic regression models were run which independently examined the association of correct responses with: (1) perceived nutrition knowledge (measured in quartiles); (2) perceived diet healthiness; and (3) dietary intake (fruit/vegetable intake and less healthy food intake). All models were adjusted for country, age, sex-at-birth, ethnicity, perceived income, and BMI. Unless otherwise indicated, all estimates are weighted, and survey weights were rescaled to the unweighted sample size of the final analytical sample. Analyses were conducted using SAS Studio v3.81 (SAS Institute Inc., North Carolina) and IBM SPSS Statistics for Windows v28.0.1.0 (IBM Corp., Armonk, New York). A STROBE checklist of items that must be included in reports of cross-sectional studies was completed.

## Results

### Demographic characteristics

The demographic profile of the sample is shown in Table 1, both overall and by country. Briefly, distributions of age and sex-at-birth were similar across all countries. The US sample had a notably higher proportion of ‘minority’ respondents and perceived income adequacy was lowest in Mexico and Chile.

**Table 1:** Demographic characteristics among the overall sample, by country, 2020 (weighted estimates, *N* = 11,232)

	Overall (*N* = 11,232) % (*N*)	Canada (*n* = 3,601) % (*n*)	Australia (*n* = 1,474) % (*n*)	UK (*n* = 1,426) % (*n*)	US (*n* = 1,509) % (*n*)	Mexico (*n* = 1,724) % (*n*)	Chile (*n* = 1,498) % (*n*)
**Age (years)**							
10–13	50.0% (5620)	50.2% (1808)	51.3% (756)	52.6% (751)	49.8% (752)	49.5% (854)	46.6% (700)
14–17	50.0% (5611)	49.8% (1793)	48.7% (717)	47.4% (675)	50.2% (757)	50.5% (870)	53.3% (798)
**Sex-at-birth**							
Male	50.9% (5713)	50.7% (1827)	51.4% (758)	50.7% (722)	51.1% (772)	50.6% (873)	50.8% (761)
Female	49.1% (5519)	49.3% (1774)	48.6% (716)	49.3% (703)	48.9% (737)	49.4% (851)	49.2% (738)
**Ethnicity†**							
Majority	73.5% (8258)	70.5% (2539)	74.5% (1098)	83.2% (1187)	51.8% (782)	80.0% (1380)	84.9% (1273)
Minority	26.5% (2974)	29.5% (1062)	25.5% (376)	16.8% (239)	48.2% (727)	20.0% (344)	15.1% (225)
**Perceived Income Adequacy**							
Not enough money	3.9% (434)	2.5% (89)	3.8% (56)	4.1% (58)	4.2% (64)	5.1% (88)	5.2% (79)
Barely enough money	20.7% (2322)	15.5% (560)	15.9% (235)	18.5% (264)	18.7% (282)	32.6% (563)	28.0% (419)
Enough money	62.5% (7025)	64.6% (2327)	64.9% (955)	64.8% (924)	59.8% (901)	57.7% (994)	61.6% (923)
More than enough money	12.9% (1451)	17.4% (626)	15.4% (228)	12.6% (180)	17.3% (261)	4.6% (79)	5.2% (78)
**BMI††**							
“Thin”	3.0% (336)	4.0% (144)	3.7% (55)	3.4% (48)	3.1% (47)	1.3% (23)	1.3% (19)
“Normal”	44.2% (4969)	51.4% (1850)	42.0% (620)	35.7% (508)	45.9% (692)	41.6% (716)	38.9% (582)
“Overweight”	17.4% (1952)	15.9% (572)	16.4% (242)	12.1% (172)	19.2% (290)	22.6% (389)	19.2% (287)
“Obese”	9.2% (1031)	8.7% (315)	8.9% (132)	6.7% (95)	12.6% (190)	10.1% (174)	8.4% (126)
Not reported	26.2% (2944)	20.0% (721)	29.0% (426)	42.1% (601)	19.2% (291)	24.5% (422)	32.2% (483)

† Majority constituted the following ethnicities: in Canada if “White (European descent)” is the only category selected; in Australia if only speak English at home; in UK and US if only the “White” category is selected; in Mexico and Chile if do not consider self Indigenous

†† BMI: Body Mass Index, wherein Thin: z-score<-2, Normal: -2 < = z-score < = 1, Overweight: 1 < z-score < = 2, Obese: z-score > 2, Not reported: don’t know/refuse to answer/extreme values (z-score<-5 or z-score > 5)

### Differences in functional nutrition knowledge by country and demographics

Table 2 shows the healthiness ratings for the apple and the fruit drink by country and demographic correlates. In terms of the “mean” healthiness rating for the apple, there was little variation across countries, with mean scores above 9 in all cases. In contrast, greater differences were observed between countries for the healthiness of the apple fruit drink, with the lowest scores in Mexico (mean = 4.63, SD = 2.77) and the highest scores in the US (mean = 6.61, SD = 2.55). The percentage of correct responses—for which respondents’ healthiness ratings were higher for the apple than the fruit drink— ranged from a low of 79.6% in the US to a high of 95.6% in Mexico. Country-specific healthiness ratings and scores for each demographic correlate are available in supplemental tables S1a-f.

**Table 2:** Healthiness ratings, by country and demographics, 2020 (weighted estimates, *N* = 11,232)

	Item Healthiness Rating Mean (SD*)		Healthiness Score			
	Apple fruit drink	Apple	% Correct**	AOR***	95% CI****	*P* value
**Country** (*N* = 11,232)						
US (*n* = 1,509)	6.61 (2.55)	9.43 (1.08)	79.6%	Ref		
UK (*n* = 1,426)	5.97 (2.31)	9.22 (1.24)	85.8%	1.58	1.28–1.97	< 0.001
Australia (*n* = 1,474)	5.16 (2.71)	9.38 (1.13)	86.2%	1.62	1.31-2.00	< 0.001
Canada (*n* = 3,601)	5.02 (2.48)	9.39 (1.05)	93.3%	3.54	2.90–4.32	< 0.001
Chile (*n* = 1,498)	5.00 (2.47)	9.39 (1.16)	94.3%	4.21	3.20–5.54	< 0.001
Mexico (*n* = 1,724)	4.63 (2.77)	9.67 (0.80)	95.6%	5.37	3.95–7.30	< 0.001
**Age (years)**						
10–13	5.53 (2.62)	9.48 (1.04)	89.3%	Ref		
14–17	5.10 (2.60)	9.36 (1.12)	90.8%	1.10	0.95–1.26	0.202
**Sex-at-birth**						
Male	5.46 (2.61)	9.42 (1.06)	88.8%	Ref		
Female	5.16 (2.62)	9.41 (1.10)	91.3%	1.29	1.13–1.49	< 0.001
**Ethnicity†**						
Majority	5.24 (2.63)	9.43 (1.07)	90.4%	Ref		
Minority	5.50 (2.60)	9.39 (1.10)	89.1%	1.05	0.88–1.24	0.612
**Perceived Income Adequacy**						
Not enough money	5.00 (2.83)	9.46 (1.20)	86.8%	Ref		
Barely enough money	5.10 (2.58)	9.38 (1.17)	91.8%	1.58	1.10–2.28	0.013
Enough money	5.34 (2.58)	9.41 (1.07)	90.6%	1.49	1.06–2.08	0.021
More than enough money	5.60 (2.78)	9.50 (0.91)	85.3%	0.98	0.68–1.41	0.925
**BMI††**						
“Normal”	5.75 (2.48)	9.50 (0.92)	90.8%	Ref		
“Thin”	5.16 (2.59)	9.43 (1.00)	91.6%	1.04	0.69–1.55	0.863
“Overweight”	5.33 (2.69)	9.39 (1.14)	89.4%	0.78	0.64–0.94	0.009
“Obese”	5.45 (2.70)	9.32 (1.19)	87.4%	0.70	0.56–0.90	0.004
Not reported	5.45 (2.61)	9.43 (1.14)	88.8%	0.76	0.64–0.90	0.002

† Majority constituted the following ethnicities: in Canada if “White (European descent)” is the only category selected; in Australia if only speak English at home; in UK and US if only the ”White” category is selected; in Mexico and Chile if do not consider self Indigenous

†† BMI: Body Mass Index, wherein Thin: z-score<-2, Normal: -2 < = z-score < = 1, Overweight: 1 < z-score < = 2, Obese: z-score > 2, Not reported: don’t know/refuse to answer/extreme values (z-score<-5 or z-score > 5)

* SD: standard deviation

** Correct = respondents rated the healthiness of the apple higher than the fruit drink

***Adjusted odds ratios from a binary logistic regression model testing differences in correct scores by country, age, sex-at-birth, ethnicity, perceived income adequacy and BMI (all included in a single model to account for confounding effects)

****95% confidence interval

In a binary logistic regression model adjusting for demographic factors, youth in the US reported a lower percentage of correct responses compared to all other countries (*p* < 0.001 for all), whereas youth in Mexico and Chile reported a higher percentage of correct responses than all other countries (*p* < 0.010 for all, except between Chile and Canada where *p* = 0.210). In addition, youth in Canada were significantly more likely to report correct responses than youth in Australia and the UK (*p* < 0.001 for both).

Differences in correct scores were also observed by sex-at-birth, income adequacy, and BMI – see Table 2. Females were more likely to have correct scores than males (*p* < 0.001), as were youth who reported having “barely enough money” and “enough money” compared to those with “not enough money” (*p* = 0.013 and *p* = 0.021, respectively). No association was observed for those who reported having “more than enough money” compared to “not enough money” (*p* = 0.925). In terms of BMI, participants in the “normal” weight status range had greater odds of a correct response compared to youth with “overweight”, “obese”, and “not reported” BMI scores (*p* < 0.010 for all), but no differences were observed for those with “thin” BMI scores (*p* = 0.863). Correct scores did not significantly differ by age and ethnicity (*p* = 0.202 and *p* = 0.612, respectively).

**Fig. 2:**
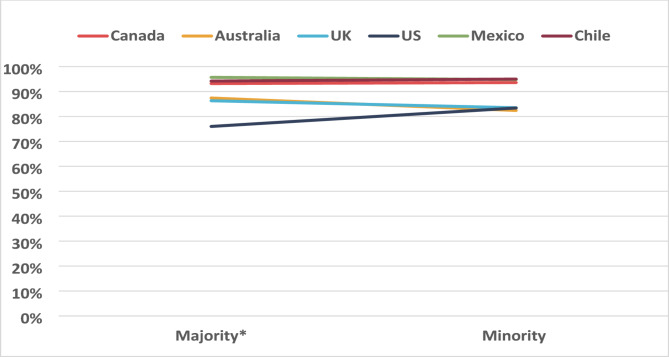
Percentage of correct responses by ethnicity and country, 2020 (weighted estimates, *N* = 11,232). * Majority constituted the following ethnicities: in Canada if “White (European descent)” is the only category selected; in Australia if only speak English at home; in UK and US if only the “White” category is selected; in Mexico and Chile if do not consider self Indigenous

A two-way interaction was observed between country and ethnicity (F value: 2.37, *p* = 0.037). As shown in Fig. 2, respondents from Australia and the UK who self-identified as ‘majority’ ethnicity were more likely to have a correct response compared to ‘minority’ respondents; in opposite, US respondents who self-identified as ‘minority’ ethnicity were more likely to have a correct response. Few differences by ethnicity were observed among youth in Canada, Mexico and Chile.

**Fig. 3:**
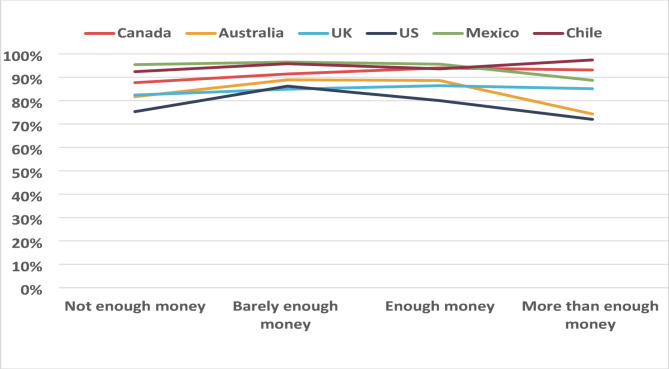
Percentage of correct responses by perceived income adequacy and country, 2020 (weighted estimates, *N* = 11,232)

A two-way interaction was also observed between country and income adequacy (F value: 2.06, *p* = 0.009). As shown in Fig. 3, in the UK, Chile, and Canada the percentage of correct scores increased with higher perceived income adequacy. In Australia and Mexico, correct responses were higher in the moderate income adequacy categories, where as US youth reporting “barely enough money” had the highest percentage of correct scores.

### Association between functional nutrition knowledge and perceived nutrition knowledge

Table 3 shows differences in perceived nutrition knowledge by country. Youth from the US and Mexico reported the highest perceived nutrition knowledge (mean = 6.86), while those from Chile reported the lowest (mean = 6.25). Chilean youth also had the highest frequency of “very low” perceived nutrition knowledge, while youth from Australia and the US reported the highest frequency of “high” perceived nutrition knowledge. Country-specific differences in perceived nutrition knowledge are additionally described for each demographic correlate in supplemental tables S1a-f.

**Table 3:** Measures of perceived nutrition knowledge, perceived diet healthiness, and diet intake, by country, 2020 (weighted estimates, *N* = 11,232)

	Overall (*N* = 11,232) % (*n*)	Canada (*n* = 3,601) % (*n*)	Australia (*n* = 1,474) % (*n*)	UK (*n* = 1,426) % (*n*)	US (*n* = 1,509) % (*n*)	Mexico (*n* = 1,724) % (*n*)	Chile (*n* = 1,498) % (*n*)
**Perceived Nutrition Knowledge**							
Mean (SD†)	6.63 (2.02)	6.61 (1.85)	6.75 (2.13)	6.41 (2.03)	6.86 (2.04)	6.86 (1.97)	6.25 (2.22)
Very Low (Quartile 1)	26.9% (3022)	24.9% (897)	26.7% (394)	29.7% (423)	24.6% (371)	22.7% (391)	36.4% (533)
Low (Quartile 2)	38.6% (4332)	43.9% (1582)	36.5% (539)	40.2% (573)	34.3% (517)	34.2% (590)	35.4% (523)
Moderate (Quartile 3)	19.7% (2208)	18.9% (682)	16.5% (243)	18.6% (265)	20.6% (311)	28.5% (490)	14.4% (214)
High (Quartile 4)	14.9% (1670)	12.2% (440)	20.2% (298)	11.5% (164)	20.5% (309)	14.6% (252)	13.8% (206)
**Perceived Diet Healthiness**							
Very unhealthy	0.9% (97)	0.5% (18)	1.2% (17)	1.1% (15)	1.5% (22)	0.6% (10)	0.9% (14)
Unhealthy	5.2% (584)	4.2% (151)	5.9% (87)	6.8% (97)	5.2% (79)	5.1% (88)	5.6% (84)
In the middle	43.8% (4921)	45.6% (1643)	41.6% (613)	53.0% (756)	46.9% (707)	35.0% (603)	39.9% (598)
Healthy	40.4% (4539)	41.7% (1503)	37.1% (547)	32.4% (462)	37.2% (561)	48.2% (831)	42.4% (636)
Very healthy	9.7% (1089)	7.9% (286)	14.2% (206)	6.7% (95)	9.3% (140)	11.1% (192)	11.1% (166)
**Fruit Intake** Mean (SD)	1.68 (1.41)	1.62 (1.37)	1.87 (1.55)	1.56 (1.38)	1.67 (1.46)	1.80 (1.35)	1.65 (1.35)
**Vegetable Intake** Mean (SD)	1.65 (1.32)	1.65 (1.26)	1.81 (1.52)	1.58 (1.34)	1.62 (1.38)	1.53 (1.21)	1.69 (1.29)
**“Less Healthy Food” Index**							
Mean (SD)	2.58 (1.25)	2.41 (1.21)	2.60 (1.33)	2.50 (1.19)	2.80 (1.28)	2.91 (1.19)	2.48 (1.25)
Sugary drinks (% yes)	50.0% (5618)	40.4% (1455)	49.5% (729)	41.5% (592)	51.4% (776)	69.9% (1205)	57.4% (860)
Fast food from Restaurant (% yes)	25.4% (2857)	23.5% (848)	38.8% (573)	21.0% (300)	39.3% (593)	18.2% (314)	15.3% (229)
Sugary cereal (% yes)	38.2% (4294)	31.0% (1116)	29.8% (439)	35.4% (504)	41.5% (626)	54.4% (939)	44.7% (670)
Snacks (% yes)	75.4% (8468)	76.5% (2755)	76.0% (1121)	78.0% (1112)	78.6% (1187)	77.9% (1343)	63.5% (951)
Dessert (% yes)	69.2% (7774)	69.1% (2489)	65.4% (964	73.6% (1050)	69.0% (1041)	70.9% (1223)	67.2% (1007)

† SD: standard deviation

In a binary logistic regression model, adjusting for the demographic variables described above, perceived nutrition knowledge was found to be significantly associated with correct scores (F value: 83.10, *p* < 0.001). As shown in Table 4, compared to all other groups, youth who reported high perceived nutrition knowledge had lower odds of correct scores, whereas those reporting low perceived nutrition knowledge had higher odds of correct scores. In particular, youth with low perceived nutrition knowledge had 3.98 times greater odds (95% CI: 3.32–4.78, *p* < 0.001) of correct scores than those reporting high perceived nutrition knowledge. The main effects of demographic correlates in this model are described in supplemental table S2; though estimates differ slightly, the pattern of findings generally remains consistent with those previously described.

**Table 4:** Healthiness ratings by perceived nutrition knowledge, perceived diet healthiness, and diet intake, 2020 (weighted estimates, *N* = 11,232)

	Healthiness Score			
	% Correct*	AOR**	95% CI***	*P* value
**Perceived Nutrition Knowledge**				
Very Low (Quartile 1)	90.6%	2.70	2.24–3.25	< 0.001
Low (Quartile 2)	93.7%	3.98	3.32–4.78	< 0.001
Moderate (Quartile 3)	91.4%	2.76	2.25–3.38	< 0.001
High (Quartile 4)	77.9%	Ref		
**Perceived Diet Healthiness**				
Very unhealthy	82.3%	1.33	0.75–2.36	0.336
Unhealthy	90.7%	2.41	1.67–3.42	< 0.001
In the middle	90.6%	2.32	1.90–2.82	< 0.001
Healthy	91.6%	2.32	1.90–2.82	< 0.001
Very healthy	81.4%	Ref		
**Diet Intake**				
Fruit intake Mean (SD†)	1.62 (1.31)	0.87	0.83–0.92	< 0.001
Vegetable intake Mean (SD)	1.58 (1.23)	0.86	0.82–0.91	< 0.001
Less healthy food index Mean (SD)	2.52 (1.22)	0.70	0.65–0.74	< 0.001

† SD: standard deviation

* Correct = respondents rated the healthiness of the apple higher than the fruit drink

**Adjusted odds ratios from three separate binary logistic regression models testing differences in correct scores by (1) perceived nutrition knowledge, (2) perceived diet healthiness, and (3) diet intake. All models were adjusted for country, age, sex-at-birth, ethnicity, perceived income adequacy and BMI

***95% confidence interval

### Association between functional nutrition knowledge and perceived diet healthiness

Table 3 shows perceived diet healthiness by country. Across all countries, most youth perceived the healthiness of their diet as “in the middle” (43.8%) or “healthy” (40.4%). Youth in the UK reported the highest levels of “very unhealthy” or “unhealthy” diets (7.9%), whereas those in Canada reported the lowest prevalence of “very unhealthy” or “unhealthy” diets (4.7%). Conversely, UK youth reported the lowest prevalence of “healthy” or “very healthy” diets (39.1%), whereas Mexican youth reported the greatest prevalence “healthy” or “very healthy” diets (59.3%). In a binary logistic regression model, adjusting for the demographic variables described above, perceived diet healthiness was found to be significantly associated with correct scores (F value: 21.77, *p* < 0.001). As shown in Table 4, youth who reported “very healthy” and “very unhealthy” diets had lower odds of correct scores compared to other groups (*p* < 0.001 for all). Correct scores did not differ between “very unhealthy” and “very healthy” diets (*p* = 0.336). Youth who perceived diet healthiness to be “in the middle” had 2.32 times greater odds of correct scores (95% CI: 1.90–2.82, *p* < 0.001) than those reporting “very healthy” and 1.75 times greater odds of correct scores (95% CI: 0.99–3.06, *p* = 0.052) than those reporting “very unhealthy” diets. The main effects of demographic correlates in this model are described in supplemental table S3; though estimates differ slightly, the pattern of findings generally remains consistent with those previously described.

### Association between functional nutrition knowledge and dietary intake

Table 3 shows differences in fruit intake, vegetable intake, and “less healthy food” intake by country. Overall, youth in the US and Mexico reported consuming more “less healthy food” compared to youth in other countries. While youth in Mexico had the highest sugary drink and sugary cereal consumption, they also reported lower fast-food consumption compared to other countries. Snack intake was similar across the countries with around three quarters of youth consuming snacks like crackers, chips or granola bars, with the exception of Chile, which had lower snack intake. Dessert consumption did not vary across countries.

In an adjusted binary logistic regression model, fruit, vegetable, and “less healthy food” intakes were all found to be significantly associated with correct scores (fruit intake: F value = 28.22, vegetable intake: F value = 30.19, “less healthy food” intake: F value = 121.93; *p* < 0.001for all). As shown in Table 4, a unit increase in fruit and vegetable intake was associated with 0.87 (95% CI: 0.83–0.92) and 0.86 (95% CI: 0.82–0.91) times lower odds of a correct score, respectively. In contrast, a unit increase on the less healthy food index was associated with 0.70 (95% CI: 0.65–0.74) times lower odds of a correct score. The main effects of demographic correlates in this model are described in supplemental table S4; though estimates differ slightly, the pattern of findings generally remains consistent with those previously described.

## Discussion

To our knowledge, the current study is among the few to directly compare nutrition knowledge of youth across multiple countries. This study has three primary findings. First, differences in functional nutrition knowledge were observed across countries and demographic groups. Second, functional nutrition knowledge was inversely associated with a measure of perceived nutrition knowledge. Third, greater functional nutrition knowledge was associated with lower intake of unhealthy foods, as well as lower intake of fruit and vegetables. These findings are discussed in more detail below.

Marked differences in functional nutrition knowledge were observed between the countries surveyed. Respondents from Mexico and Chile had the highest odds of correctly identifying a fresh fruit as healthier than a SSB (the apple fruit drink). Differences in correct scores were primarily due to different impressions of the fruit drink: the apple was almost universally identified as extremely healthy, whereas the fruit drink had higher healthiness ratings in the US and relatively lower healthiness ratings in Mexico and Chile. Notably, Mexico and Chile are the only countries out of those surveyed to have mandatory front-of-pack labelling policies [52] in which SSBs, like the fruit drink shown in the current study, must display a black octagon “high-in” warning symbol on the front of packages based on high added sugar content. Previous studies have shown that these warning labels lower perceived healthiness of foods, including among young people [53, 54]. Chile and Mexico also have more comprehensive restrictions on marketing of unhealthy foods and drinks to youth, which may, in combination with labeling policies, reinforce attitudes around perceived healthiness of SSBs [55, 56]. As such, differences in perceived fruit-drink healthiness are likely the main contributor to differences in correct scores, given the universal rating of the apple. Future research should examine whether mandated front-of-pack labelling policies impact functional nutrition knowledge in a way that extends beyond the labeled products to influence perceptions across product categories more broadly; for example, whether the inclusion of “high-in” warning symbols on SSBs impact perceived healthiness for other drinks which do not have the warning symbols such as milk.

Some differences in functional nutrition knowledge were also observed based on demographic characteristics. Namely, female respondents had higher odds of correct scores, while respondents with higher BMI scores had lower odds of correct scores. Similar trends have been observed in the HELENA Study, in which adolescent girls had higher functional nutrition knowledge than boys, with a greater tendency for lower scores reported with increasing BMI [41]. Gender-differences may be related to the tendency for girls to be more ‘health conscious’ and attach greater importance to diet [57–60], in part due to the internalization of weight ideals [61, 62], which may result in increased functional nutrition knowledge as girls seek out and are exposed to more nutrition information. Literature on the associations between nutrition knowledge and BMI for children and adolescents appears to be mixed, likely due to a potentially wide range of other factors associated with BMI (such as socio-economic status) [63–66]. No significant differences by age were observed in the present study, consistent with the HELENA study and other literature which demonstrates weak associations [5, 13, 67, 68]. The present study also found some differences based on income adequacy; however, the magnitude of these differences was modest in many cases and more mixed compared with other studies that demonstrate strong associations between higher socioeconomic status and increased functional nutrition knowledge [1, 11, 69].

With respect to functional and perceived nutrition knowledge, there was an inverse association. Respondents who believed themselves to possess the highest levels of nutrition knowledge were less likely to correctly rank the items and vice versa – indicating a discrepancy in self-reported and objective knowledge. To our knowledge, few studies have compared perceived and functional measures of nutrition knowledge. One study conducted in Barcelona among athletes aged 13–25 found that lower scores on an objective measure of nutrition knowledge were associated with higher self-estimations of nutrition knowledge [70]. Similar findings have been observed among adult populations, in which a majority of participants over-estimated their nutrition knowledge when compared with an objective measure, including in a study using an adult version of the current study’s functional knowledge measure [71, 72]. This pattern has also been identified in other public health areas, with functional knowledge not (or very weakly) associated with perceived knowledge [73, 74]. This aligns with the general principle described as the ‘Dunning Kruger effect’, wherein individuals who score poorer on knowledge tests tend to overestimate their performance, while improved performance is associated with a greater recognition of knowledge limitations [75]. The Dunning Kruger effect has been observed in the nutrition context, with individuals possessing greater functional nutrition knowledge (measured as the ability to identify the nutrition score of an advertised food product) more likely to underestimate knowledge when self-reporting [76]. The reason for this effect is not clear and may be related to various demographic factors. In the current study, youth from the US reported the highest level of perceived nutrition knowledge, but the lowest level of correct scores, whereas the reverse was true in Chile. Other notable discrepancies between functional and perceived measures were observed within countries: among youth from the US, those identifying as a “minority” ethnicity had higher levels of correct scores, but lower self-reported nutrition knowledge (see supplemental table S1d). Further research is needed to understand the extent to which cultural differences may account for the observed differences in self-reported and objective nutrition knowledge, particularly as they relate to confidence in one’s own abilities or knowledge level. Some research has explored cultural differences in positive self-evaluation, identifying that those from more individualistic cultures may appraise themselves more positively than those from collectivist cultures, potentially due to cultural differences in modesty [77, 78]. However, most research on this topic has compared East Asian and Western cultures, and further research is needed exploring how culture may influence self-reporting of nutrition knowledge specifically. Further, while perceived and functional measures of nutrition knowledge commonly align for experts, functional measures tend to provide a more accurate estimate for non-expert respondents, such as the youth in the current study [79]. Overall, findings from the current study support greater use of functional measures of nutrition knowledge.

Finally, we found mixed results on the association between correct scores and self-reported diet intake. Correct responses were associated with lower intake of less healthy foods; however, in contrast to our hypotheses, correct responses were also associated with lower self-reported intake of fruit and vegetables. In other literature, functional nutrition knowledge tends to be positively associated with increased fruit and vegetable consumption and decreased unhealthy food consumption [1, 11, 14, 69, 70]. It is possible that children and youth with lower fruit and vegetable intake were more familiar with the unhealthy profile of SSBs due to greater SSB consumption. However, this interpretation is speculative, and further research is required to replicate this finding; future research should consider using multiple 24-hour recalls or food frequency questionnaires to provide a more comprehensive assessment of fruit and vegetable intake when investigating associations with functional nutrition knowledge. Additionally, research conducted with Canadian youth has identified more severe household food insecurity to be associated with greater ultra-processed food consumption and lower diet quality; no significant trends in fruit/vegetable intake were observed [80]. Given the universal high rating of the apple, greater consumption of SSBs may have resulted in the SSBs being scored more favorably, thus increasing likelihood of incorrect scores among youth with greater food insecurity. Further research is needed to examine the potential for food insecurity to moderate the relationship between dietary intake and functional nutrition knowledge.

### Limitations

The present study has limitations which are common to survey research. Recruitment of respondents involved nonprobability-based sampling, which limits the national representativeness of the findings. Nevertheless, quota sampling and post-stratification weights were created for each country based on sex-at-birth, region, age and ethnicity (except in Canada). The prevalence of self-reported “overweight” and “obesity” BMI scores was also comparable with each country’s national benchmark surveys [44].

Further, an aggregated measure of ethnicity was used in the current analyses to accommodate differences in the way that ethnicity is assessed across countries, as IFPS Youth surveys use ethnicity questions from country-specific national census or ‘gold standard’ health surveys. As such, detailed interpretation is limited, and further studies are needed to examine country-specific differences in functional and perceived nutrition knowledge across ethnicities in greater depth. The present study also has a cross-sectional survey design; longitudinal studies are needed to provide further context on the trends in knowledge and association with dietary intake. Additionally, as mentioned above, front-of-pack labels in the shape of black octagons were present on the apple fruit drink for surveys administered in Mexico and Chile, but not other countries. While a potential source of inconsistency, this was done to mimic the genuine consumer environment and display of the items as youth would normally encounter them. Finally, the current study tested only one example of an unprocessed versus ultra-processed food. The apple and SSB were selected on the basis that they are common foods among children and adolescents; however, the findings should not be generalized to the wide range of different ultra-processed foods. Further research among youth should examine a broader range of food products across processing levels for a more comprehensive assessment.

### Implications for research and practice

Across countries, between 5 and 20% of youth are unable to distinguish between the nutritional quality of a fresh fruit and an SSB—a very basic test of nutrition knowledge based on unprocessed versus ultra-processed foods. The extent to which different levels of knowledge between countries reflect national-level nutrition policies, such as front-of-pack labelling, warrants further attention. Further, education campaigns are needed to ensure that young people have basic knowledge about the relative dietary quality of commonly consumed foods, particularly in the US. The findings also suggest that self-reported nutrition knowledge is not a reliable indicator of objectively assessed nutrition knowledge in terms of the ability to correctly identify the relative healthiness of ultra-processed versus unprocessed foods among youth. This finding has implications for other studies that rely on self-reported nutrition knowledge and highlights the need for functional measures of knowledge that can be included in population-level surveys. Enhanced use of functional knowledge measures has the potential to advance our understanding of the ways in which knowledge is associated with dietary intake, as well as ‘upstream’ nutrition policies. Cross-country comparisons provide an important setting in which to conduct this work given the potential to assess “natural experiments” in nutrition policies and food environments.

## Electronic supplementary material

Below is the link to the electronic supplementary material.

Supplementary Material 1

## Data Availability

The datasets used and analysed during the current study are available from the corresponding author on reasonable request.

## Abbreviations

BMI: Body Mass Index
HELENA: Healthy Lifestyle in Europe by Nutrition in Adolescence study
IFPS: International Food Policy Study
SSB: Sugar-Sweetened Beverage
UK: United Kingdom
US: United States
