# Supplementary material for: Can kids identify unprocessed fruit as healthier than an ultra-processed sugar-sweetened beverage? Functional versus self-reported nutrition knowledge and dietary intake among youth from six countries: findings from the International Food Policy Study
Source: BMC Nutr. 2025 Jul 1;11:115. doi: 10.1186/s40795-025-01109-y (PMC12220526; doi:10.1186/s40795-025-01109-y)
Supplement: Supplementary file 1 — Supplementary Material 1 [file 40795_2025_1109_MOESM1_ESM.docx]

**Supplemental Table S1a.** Measures of nutrition knowledge and socio-demographic characteristics in Canada, 2020 (weighted estimates, N=11,232)

| Canada  (n=3,601) | **Item** **Healthiness Rating**  mean (SD) | | **Healthiness Score**  % (n) | | **Perceived**  **Nutrition Knowledge**  mean (SD) |
| --- | --- | --- | --- | --- | --- |
|  | Apple | Apple Fruit Drink | 0/1 | 1/1 |  |
| **Age (years)** |  |  |  |  |  |
| 10-13 | 9.45 (1.03) | 5.20  (2.49) | 6.9% (125) | 93.1% (1683) | 6.59 (1.83) |
| 14-17 | 9.33 (1.07) | 4.84  (2.46) | 6.5% (116) | 93.5% (1677) | 6.63 (1.87) |
| **Sex-at-birth** |  |  |  |  |  |
| Male | 9.35 (1.06) | 5.12  (2.49) | 7.7% (141) | 92.3% (1686) | 6.48 (1.89) |
| Female | 9.43 (1.04) | 4.91  (2.48) | 5.6% (100) | 94.4% (1674) | 6.75 (1.79) |
| **Ethnicity** |  |  |  |  |  |
| Majority | 9.42 (1.02) | 4.93  (2.50) | 6.8% (174) | 93.2% (2366) | 6.57 (1.85) |
| Minority | 9.33 (1.12) | 5.23  (2.43) | 6.4% (67) | 93.6% (994) | 6.71 (1.83) |
| **Perceived Income Adequacy** |  |  |  |  |  |
| Not enough money | 9.43 (1.15) | 4.74  (2.79) | 12.3% (11) | 87.7% (78) | 6.71 (2.04) |
| Barely enough  money | 9.24 (1.32) | 5.18  (2.54) | 8.6% (48) | 91.4% (512) | 6.41 (1.93) |
| Enough money | 9.41 (1.00) | 5.03  (2.45) | 6.0% (139) | 94.0% (2188) | 6.56 (1.80) |
| More than enough  money | 9.44 (0.93) | 4.87  (2.49) | 6.9% (43) | 93.1% (582) | 6.97 (1.87) |
| **BMI** |  |  |  |  |  |
| “Normal” | 9.48 (0.88) | 5.62  (2.19) | 5.8%  (8) | 94.2% (136) | 6.88 (1.66) |
| “Thin” | 9.41 (0.99) | 4.88  (2.46) | 5.1% (95) | 94.9% (1755) | 6.75 (1.77) |
| “Overweight” | 9.36 (1.18) | 4.95  (2.56) | 7.7% (44) | 92.3% (527) | 6.50 (1.84) |
| “Obese” | 9.23 (1.17) | 5.21  (2.46) | 11.7% (37) | 88.3% (278) | 6.36 (2.11) |
| Not reported | 9.43 (1.09) | 5.24  (2.52) | 7.8% (57) | 92.2% (664) | 6.42 (1.90) |

**Supplemental Table S1b**. Measures of nutrition knowledge and socio-demographic characteristics in Australia, 2020 (weighted estimates, N=11,232)

| Australia  (n=1,474) | **Item** **Healthiness Rating**  mean (SD) | | **Healthiness Score**  % (n) | | **Perceived**  **Nutrition Knowledge**  mean (SD) |
| --- | --- | --- | --- | --- | --- |
|  | Apple | Apple Fruit Drink | 0/1 | 1/1 |  |
| **Age (years)** |  |  |  |  |  |
| 10-13 | 9.51  (0.92) | 5.49  (2.70) | 14.7% (112) | 85.3% (645) | 6.91 (2.18) |
| 14-17 | 9.24 (1.31) | 4.82  (2.69) | 12.9% (93) | 87.1% (625) | 6.58 (2.05) |
| **Sex-at-birth** |  |  |  |  |  |
| Male | 9.37  (1.09) | 5.33  (2.71) | 16.0% (121) | 84.0% (637) | 6.65 (2.23) |
| Female | 9.39 (1.17) | 4.98  (2.71) | 11.6% (83) | 88.4% (633) | 6.86 (2.00) |
| **Ethnicity** |  |  |  |  |  |
| Majority | 9.38 (1.12) | 5.00  (2.70) | 12.6% (128) | 87.4% (960) | 6.65 (2.11) |
| Minority | 9.37 (1.16) | 5.64  (2.69) | 17.6% (66) | 82.4% (310) | 7.03 (2.15) |
| **Perceived Income Adequacy** |  |  |  |  |  |
| Not enough money | 9.14 (1.98) | 5.30  (2.66) | 18.3% (10) | 81.7% (46) | 6.01 (2.31) |
| Barely enough  money | 9.30  (1.22) | 4.91  (2.39) | 11.1% (26) | 88.9% (209) | 6.16 (2.29) |
| Enough money | 9.36 (1.12) | 4.98  (2.67) | 11.4% (109) | 88.6% (846) | 6.70 (1.98) |
| More than enough  money | 9.61 (0.70) | 6.16  (3.02) | 25.7% (59) | 74.3% (169) | 7.74 (2.16) |
| **BMI** |  |  |  |  |  |
| “Normal” | 9.55 (0.70) | 5.40  (2.82) | 11.2% (6) | 88.8% (49) | 7.53 (1.87) |
| “Thin” | 9.35 (1.11) | 5.07  (2.70) | 13.9% (86) | 86.1% (534) | 7.04 (1.90) |
| “Overweight” | 9.31 (1.18) | 5.44  (2.83) | 15.0% (36) | 85.0% (206) | 6.63 (2.30) |
| “Obese” | 9.24 (1.30) | 5.29  (2.90) | 18.1% (24) | 81.9% (108) | 6.50 (2.25) |
| Not reported | 9.48 (1.12) | 5.07  (2.60) | 12.2% (52) | 87.8% (374) | 6.37 (2.25) |

**Supplemental Table S1c**. Measures of nutrition knowledge and socio-demographic characteristics in the United Kingdom, 2020 (weighted estimates, N=11,232)

| UK  (n=1,426) | **Item** **Healthiness Rating**  mean (SD) | | **Healthiness Score**  % (n) | | **Perceived**  **Nutrition Knowledge**  mean (SD) |
| --- | --- | --- | --- | --- | --- |
|  | Apple | Apple Fruit Drink | 0/1 | 1/1 |  |
| **Age (years)** |  |  |  |  |  |
| 10-13 | 9.22 (1.28) | 6.05  (2.31) | 15.1% (113) | 84.9% (638) | 6.47 (2.02) |
| 14-17 | 9.22 (1.20) | 5.89  (2.30) | 13.2% (89) | 86.8% (586) | 6.34 (2.05) |
| **Sex-at-birth** |  |  |  |  |  |
| Male | 9.29 (1.14) | 6.11  (2.27) | 14.6% (106) | 85.4% (617) | 6.41 (2.05) |
| Female | 9.16 (1.33) | 5.83  (2.34) | 13.7% (97) | 86.3% (607) | 6.41 (2.01) |
| **Ethnicity** |  |  |  |  |  |
| Majority | 9.20 (1.28) | 5.96  (2.27) | 13.7% (163) | 86.3% (1024) | 6.33 (2.04) |
| Minority | 9.32 (1.06) | 6.03  (2.48) | 16.5% (40) | 83.5% (200) | 6.77 (1.94) |
| **Perceived Income Adequacy** |  |  |  |  |  |
| Not enough money | 9.30 (1.27) | 5.79  (2.39) | 17.6%  (10) | 82.4% (48) | 5.74 (2.36) |
| Barely enough  money | 9.23 (1.32) | 6.07  (2.40) | 15.1%  (40) | 84.9% (224) | 6.32 (2.17) |
| Enough money | 9.18 (1.25) | 5.96  (2.21) | 13.6% (126) | 86.4% (799) | 6.38 (1.96) |
| More than enough  money | 9.37 (1.03) | 5.94  (2.61) | 14.9% (27) | 85.1% (153) | 6.88 (2.02) |
| **BMI** |  |  |  |  |  |
| “Normal” | 9.39 (0.98) | 6.17  (2.45) | 10.8%  (5) | 89.2% (43) | 6.67 (2.04) |
| “Thin” | 9.24 (1.13) | 5.78  (2.31) | 11.3% (58) | 88.7% (451) | 6.62 (1.85) |
| “Overweight” | 9.26 (1.16) | 5.90  (2.26) | 14.0% (24) | 86.0% (148) | 6.65 (2.00) |
| “Obese” | 8.87 (1.60) | 5.78  (2.05) | 14.9% (14) | 85.1% (81) | 5.85 (2.24) |
| Not reported | 9.24 (1.30) | 6.17  (2.33) | 16.8% (101) | 83.2% (500) | 6.23 (2.12) |

**Supplemental Table S1d**. Measures of nutrition knowledge and socio-demographic characteristics in United States, 2020 (weighted estimates, N=11,232)

| US  (n=1,509) | **Item** **Healthiness Rating**  mean (SD) | | **Healthiness Score**  % (n) | | **Perceived**  **Nutrition Knowledge**  mean (SD) |
| --- | --- | --- | --- | --- | --- |
|  | Apple | Apple Fruit Drink | 0/1 | 1/1 |  |
| **Age (years)** |  |  |  |  |  |
| 10-13 | 9.53 (0.98) | 6.96  (2.52) | 23.1% (174) | 76.9% (578) | 6.85 (2.07) |
| 14-17 | 9.32  (1.16) | 6.26  (2.53) | 17.8% (135) | 82.2% (623) | 6.87 (2.01) |
| **Sex-at-birth** |  |  |  |  |  |
| Male | 9.46 (1.09) | 6.86  (2.53) | 24.0% (185) | 76.0% (586) | 6.89 (2.13) |
| Female | 9.39 (1.07) | 6.35  (2.55) | 16.6% (123) | 83.4% (615) | 6.83 (1.94) |
| **Ethnicity** |  |  |  |  |  |
| Majority | 9.46 (1.05) | 7.01  (2.47) | 24.0% (188) | 76.0% (594) | 6.99 (1.98) |
| Minority | 9.38 (1.11) | 6.18  (2.56) | 16.6% (121) | 83.4% (607) | 6.72 (2.09) |
| **Perceived Income Adequacy** |  |  |  |  |  |
| Not enough money | 9.43 (1.12) | 5.85  (2.92) | 24.7% (16) | 75.3% (48) | 5.76 (2.37) |
| Barely enough  money | 9.33 (1.09) | 6.00  (2.62) | 13.8% (39) | 82.6% (243) | 6.53 (1.97) |
| Enough money | 9.41 (1.13) | 6.78  (2.40) | 20.0% (180) | 80.0% (721) | 6.89 (1.92) |
| More than enough  money | 9.57 (0.86) | 6.89  (2.73) | 28.0% (73) | 72.0% (188) | 7.39 (2.26) |
| **BMI** |  |  |  |  |  |
| “Normal” | 9.48 (0.82) | 6.52  (2.13) | 17.6% (8) | 82.4% (38) | 7.03 (2.02) |
| “Thin” | 9.42 (1.02) | 6.50  (2.51) | 18.9% (131) | 81.1% (562) | 7.12 (1.85) |
| “Overweight” | 9.48 (0.99) | 7.05  (2.34) | 24.3% (70) | 75.7% (219) | 7.08 (1.97) |
| “Obese” | 9.43 (1.11) | 6.66  (2.69) | 20.6% (39) | 79.4% (151) | 6.41 (1.99) |
| Not reported | 9.38 (1.30) | 6.42  (2.75) | 20.7% (60) | 79.3% (230) | 6.28 (2.38) |

**Supplemental Table S1e**. Measures of nutrition knowledge and socio-demographic characteristics in Mexico, 2020 (weighted estimates, N=11,232)

| Mexico  (n=1,724) | **Item** **Healthiness Rating**  mean (SD) | | **Healthiness Score**  % (n) | | **Perceived**  **Nutrition Knowledge**  mean (SD) |
| --- | --- | --- | --- | --- | --- |
|  | Apple | Apple Fruit Drink | 0/1 | 1/1 |  |
| **Age (years)** |  |  |  |  |  |
| 10-13 | 9.69 (0.79) | 4.75  (2.73) | 4.5% (38) | 95.5% (815) | 6.96 (1.84) |
| 14-17 | 9.66 (0.12) | 4.52  (2.80) | 4.4% (38) | 95.6% (832) | 6.75 (2.08) |
| **Sex-at-birth** |  |  |  |  |  |
| Male | 9.67 (0.84) | 4.74  (2.77) | 4.5% (39) | 95.5% (833) | 6.81 (1.98) |
| Female | 9.68 (0.77) | 4.52  (2.76) | 4.4% (37) | 95.6% (814) | 6.90 (1.96) |
| **Ethnicity** |  |  |  |  |  |
| Majority | 9.67 (0.79) | 4.59  (2.74) | 4.3% (59) | 95.7% (1321) | 6.87 (1.90) |
| Minority | 9.67 (0.86) | 4.78  (2.86) | 5.2% (18) | 94.8% (327) | 6.79 (2.23) |
| **Perceived Income Adequacy** |  |  |  |  |  |
| Not enough money | 9.73 (0.71) | 4.08  (2.99) | 4.6%  (4) | 95.4% (84) | 5.98 (2.41) |
| Barely enough  money | 9.66 (0.79) | 4.45  (2.64) | 3.5%  (20) | 96.5% (543) | 6.56 (1.99) |
| Enough money | 9.68 (0.78) | 4.76  (2.79) | 4.4% (44) | 95.6% (950) | 7.06 (1.86) |
| More than enough  money | 9.64 (1.15) | 4.97  (3.06) | 11.3% (9) | 88.7% (70) | 7.45 (2.06) |
| **BMI** |  |  |  |  |  |
| “Normal” | 9.77 (0.53) | 4.76  (2.57) | 7.3%  (2) | 92.7% (21) | 7.41 (2.03) |
| “Thin” | 9.66 (0.77) | 4.43  (2.71) | 4.3% (31) | 95.7% (658) | 7.05 (1.74) |
| “Overweight” | 9.61 (0.96) | 4.68  (2.67) | 3.5%  (14) | 96.5% (376) | 6.96 (1.84) |
| “Obese” | 9.70 (0.74) | 4.87  (3.11) | 3.4%  (6) | 96.6% (168) | 6.67 (2.14) |
| Not reported | 9.74 (0.74) | 4.82  (2.74) | 5.8%  (25) | 94.2% (397) | 6.48 (2.30) |

**Supplemental Table S1f**. Measures of nutrition knowledge and socio-demographic characteristics in Chile, 2020 (weighted estimates, N=11,232)

| Chile  (n=1,498) | **Item** **Healthiness Rating**  mean (SD) | | **Healthiness Score**  % (n) | | **Perceived**  **Nutrition Knowledge**  mean (SD) |
| --- | --- | --- | --- | --- | --- |
|  | Apple | Apple Fruit Drink | 0/1 | 1/1 |  |
| **Age (years)** |  |  |  |  |  |
| 10-13 | 9.44 (1.18) | 5.25  (2.48) | 5.5% (38) | 94.5% (661) | 6.14 (2.22) |
| 14-17 | 9.34 (1.14) | 4.78  (2.44) | 5.8% (46) | 94.2% (752) | 6.35 (2.22) |
| **Sex-at-birth** |  |  |  |  |  |
| Male | 9.43 (1.11) | 5.18  (2.38) | 6.1% (46) | 93.9% (714) | 6.25 (2.20) |
| Female | 9.34 (1.21) | 4.81  (2.55) | 5.2% (39) | 94.8% (699) | 6.25 (2.25) |
| **Ethnicity** |  |  |  |  |  |
| Majority | 9.40 (1.15) | 5.01  (2.48) | 5.8% (73) | 94.2% (1199) | 6.32 (2.17) |
| Minority | 9.33 (1.21) | 4.91  (2.39) | 5.0%  (11) | 95.0% (214) | 5.86 (2.44) |
| **Perceived Income Adequacy** |  |  |  |  |  |
| Not enough money | 9.58 (0.91) | 4.82  (2.72) | 7.6%  (6) | 92.4% (73) | 5.26 (2.90) |
| Barely enough  money | 9.34 (1.24) | 4.77  (2.42) | 4.2%  (18) | 95.8% (401) | 5.75 (2.14) |
| Enough money | 9.37 (1.17) | 5.09  (2.47) | 6.4% (59) | 93.6% (864) | 6.51 (2.14) |
| More than enough  money | 9.59 (0.82) | 5.26  (2.43) | 2.6%  (2) | 97.4% (76) | 6.94 (2.08) |
| **BMI** |  |  |  |  |  |
| “Normal” | 9.57 (1.79) | 6.07  (2.44) | 7.3%  (1) | 92.7% (18) | 5.89 (2.51) |
| “Thin” | 9.48 (0.98) | 4.93  (2.37) | 3.2%  (18) | 96.8% (564) | 6.62 (2.11) |
| “Overweight” | 9.19 (1.32) | 4.79  (2.61) | 6.6%  (19) | 93.4% (268) | 6.24 (2.07) |
| “Obese” | 9.23 (1.27) | 4.96  (2.33) | 8.1%  (10) | 91.9% (116) | 5.53 (2.27) |
| Not reported | 9.42 (1.18) | 5.17  (2.52) | 7.4% (36) | 92.6% (447) | 6.02 (2.34) |

**Supplemental Table S2**: Main effects model of healthiness score by perceived nutrition knowledge, 2020 (weighted estimates, N=11,232)

|  | **Healthiness Score** | | | | |
| --- | --- | --- | --- | --- | --- |
|  |  |  | **1 vs 0** | | |
|  | **% 0/1** | **% 1/1** | **AOR*** | **95% CI**** | **P value** |
| **Country** |  |  |  |  |  |
| US (n=1,509) | 20.4% | 79.6% | Ref |  |  |
| UK (n=1,426) | 14.2% | 85.8% | 1.44 | 1.15-1.79 | 0.001 |
| Canada (n=3,601) | 6.7% | 93.3% | 3.20 | 2.62-3.92 | <.001 |
| Australia (n=1,474) | 13.8% | 86.2% | 1.66 | 1.35-2.04 | <.001 |
| Mexico (n=1,724) | 4.4% | 95.6% | 5.44 | 3.99-7.41 | <.001 |
| Chile (n=1,498) | 5.7% | 94.3% | 4.18 | 3.16-5.54 | <.001 |
| **Age (years)** |  |  |  |  |  |
| 10-13 | 10.7% | 89.3% | Ref |  |  |
| 14-17 | 9.2% | 90.8% | 1.05 | 0.91-1.21 | 0.487 |
| **Sex-at-birth** |  |  |  |  |  |
| Male | 11.2% | 88.8% | Ref |  |  |
| Female | 8.7% | 91.3% | 1.29 | 1.12-1.49 | <.001 |
| **Ethnicity** |  |  |  |  |  |
| Majority | 9.6% | 90.4% | Ref |  |  |
| Minority | 10.9% | 89.1% | 1.09 | 0.92- 1.29 | 0.323 |
| **Perceived Income Adequacy** |  |  |  |  |  |
| Not enough money | 13.2% | 86.8% | Ref |  |  |
| Barely enough  money | 8.2% | 91.8% | 1.51 | 1.04-2.20 | 0.029 |
| Enough money | 9.4% | 90.6% | 1.47 | 1.04-2.07 | 0.030 |
| More than enough  money | 14.7% | 85.3% | 1.15 | 0.80-1.67 | 0.450 |
| **BMI** |  |  |  |  |  |
| “Normal” | 9.2% | 90.8% | Ref |  |  |
| “Thin” | 8.4% | 91.6% | 1.10 | 0.73-1.67 | 0.650 |
| “Overweight” | 10.6% | 89.4% | 0.76 | 0.63-0.93 | 0.007 |
| “Obese” | 12.6% | 87.4% | 0.68 | 0.53-0.86 | 0.002 |
| Not reported | 11.2% | 88.8% | 0.73 | 0.61-0.86 | <.001 |
| **Perceived**  **Nutrition Knowledge** |  |  |  |  |  |
| Very Low  (Quartile 1) | 9.4% | 90.6% | 2.70 | 2.24-3.25 | <.001 |
| Low  (Quartile 2) | 6.3% | 93.7% | 3.98 | 3.32-4.78 | <.001 |
| Moderate  (Quartile 3) | 8.6% | 91.4% | 2.76 | 2.25-3.38 | <.001 |
| High  (Quartile 4) | 22.1% | 77.9% | Ref |  |  |

*Odds ratio from a binary logistic regression model adjusted for country, age, sex-at-birth, ethnicity, perceived income adequacy and BMI.

**95% confidence interval

**Supplemental Table S3**: Main effects model of healthiness score by perceived diet healthiness, 2020 (weighted estimates, N=11,232)

|  | **Healthiness Score** | | | | |
| --- | --- | --- | --- | --- | --- |
|  |  |  | **1 vs 0** | | |
|  | **% 0/1** | **% 1/1** | **AOR*** | **95% CI**** | **P value** |
| **Country** |  |  |  |  |  |
| US (n=1,509) | 20.4% | 79.6% | Ref |  |  |
| UK (n=1,426) | 14.2% | 85.8% | 1.56 | 1.25-1.93 | <.001 |
| Canada (n=3,601) | 6.7% | 93.3% | 3.49 | 2.86-4.27 | <.001 |
| Australia (n=1,474) | 13.8% | 86.2% | 1.72 | 1.39-2.12 | <.001 |
| Mexico (n=1,724) | 4.4% | 95.6% | 5.67 | 4.15-7.74 | <.001 |
| Chile (n=1,498) | 5.7% | 94.3% | 4.45 | 3.36-5.88 | <.001 |
| **Age (years)** |  |  |  |  |  |
| 10-13 | 10.7% | 89.3% | Ref |  |  |
| 14-17 | 9.2% | 90.8% | 1.07 | 0.93-1.23 | 0.336 |
| **Sex-at-birth** |  |  |  |  |  |
| Male | 11.2% | 88.8% | Ref |  |  |
| Female | 8.7% | 91.3% | 1.28 | 1.11-1.47 | 0.001 |
| **Ethnicity** |  |  |  |  |  |
| Majority | 9.6% | 90.4% | Ref |  |  |
| Minority | 10.9% | 89.1% | 1.05 | 0.89-1.25 | 0.541 |
| **Perceived Income Adequacy** |  |  |  |  |  |
| Not enough money | 13.2% | 86.8% | Ref |  |  |
| Barely enough  money | 8.2% | 91.8% | 1.54 | 1.07-2.21 | 0.019 |
| Enough money | 9.4% | 90.6% | 1.50 | 1.08-2.10 | 0.017 |
| More than enough  money | 14.7% | 85.3% | 1.14 | 0.79-1.63 | 0.492 |
| **BMI** |  |  |  |  |  |
| “Normal” | 9.2% | 90.8% | Ref |  |  |
| “Thin” | 8.4% | 91.6% | 1.06 | 0.71-1.57 | 0.778 |
| “Overweight” | 10.6% | 89.4% | 0.77 | 0.64-0.94 | 0.010 |
| “Obese” | 12.6% | 87.4% | 0.68 | 0.54-0.87 | 0.002 |
| Not reported | 11.2% | 88.8% | 0.75 | 0.63-0.89 | 0.001 |
| **Diet Healthiness** |  |  |  |  |  |
| Very unhealthy | 17.7% | 82.3% | 1.33 | 0.75-2.36 | 0.3358 |
| Unhealthy | 9.3% | 90.7% | 2.41 | 1.67-3.42 | <.001 |
| In the middle | 9.4% | 90.6% | 2.32 | 1.90-2.82 | <.001 |
| Healthy | 8.4% | 91.6% | 2.32 | 1.90-2.82 | <.001 |
| Very healthy | 18.6% | 81.4% | Ref |  |  |

*Odds ratio from a binary logistic regression model adjusted for country, age, sex-at-birth, ethnicity, perceived income adequacy and BMI.

**95% confidence interval

**Supplemental Table S4**: Main effects model of healthiness score by dietary intake, 2020 (weighted estimates, N=11,232)

|  | **Healthiness Score** | | | | |
| --- | --- | --- | --- | --- | --- |
|  |  |  | **1 vs 0** | | |
|  | **% 0/1** | **% 1/1** | **AOR*** | **95% CI**** | **P value** |
| **Country** |  |  |  |  |  |
| US (n=1,509) | 20.4% | 79.6% | Ref |  |  |
| UK (n=1,426) | 14.2% | 85.8% | 1.40 | 1.12-1.75 | 0.003 |
| Canada (n=3,601) | 6.7% | 93.3% | 3.12 | 2.54-3.83 | <.001 |
| Australia (n=1,474) | 13.8% | 86.2% | 1.67 | 1.35-2.06 | <.001 |
| Mexico (n=1,724) | 4.4% | 95.6% | 6.04 | 4.43-8.24 | <.001 |
| Chile (n=1,498) | 5.7% | 94.3% | 4.10 | 3.10-5.43 | <.001 |
| **Age (years)** |  |  |  |  |  |
| 10-13 | 10.7% | 89.3% | Ref |  |  |
| 14-17 | 9.2% | 90.8% | 1.00 | 0.87-1.16 | 0.975 |
| **Sex-at-birth** |  |  |  |  |  |
| Male | 11.2% | 88.8% | Ref |  |  |
| Female | 8.7% | 91.3% | 1.24 | 1.08-1.43 | 0.003 |
| **Ethnicity** |  |  |  |  |  |
| Majority | 9.6% | 90.4% | Ref |  |  |
| Minority | 10.9% | 89.1% | 1.06 | 0.90-1.26 | 0.480 |
| **Perceived Income Adequacy** |  |  |  |  |  |
| Not enough money | 13.2% | 86.8% | Ref |  |  |
| Barely enough  money | 8.2% | 91.8% | 1.63 | 1.12-2.39 | 0.012 |
| Enough money | 9.4% | 90.6% | 1.60 | 1.13-2.28 | 0.009 |
| More than enough  money | 14.7% | 85.3% | 1.28 | 0.88-1.88 | 0.197 |
| **BMI** |  |  |  |  |  |
| “Normal” | 9.2% | 90.8% | Ref |  |  |
| “Thin” | 8.4% | 91.6% | 1.09 | 0.72-1.65 | 0.673 |
| “Overweight” | 10.6% | 89.4% | 0.83 | 0.68-1.01 | 0.060 |
| “Obese” | 12.6% | 87.4% | 0.76 | 0.59-0.97 | 0.029 |
| Not reported | 11.2% | 88.8% | 0.76 | 0.64-0.90 | 0.002 |
| **Diet Intake** |  |  |  |  |  |
| Fruit intake  mean (SD) | 2.28 (1.96) | 1.62  (1.31) | 0.87 | 0.83-0.92 | <.001 |
| Vegetable intake  mean (SD) | 2.22 (1.89) | 1.58  (1.23) | 0.86 | 0.82-0.91 | <.001 |
| Less healthy food  Index  mean (SD) | 3.15 (1.38) | 2.52  (1.22) | 0.70 | 0.65-0.74 | <.001 |

*Odds ratio from a binary logistic regression model adjusted for country, age, sex-at-birth, ethnicity, perceived income adequacy and BMI.

**95% confidence interval
